# The incidence and outcomes of traumatic cauda equina syndrome in Victoria, Australia

**DOI:** 10.1016/j.xnsj.2024.100558

**Published:** 2024-09-12

**Authors:** Xenia Zubenko, Susan Liew, Sandra Reeder, Yi Yang, Ali Humadi, Belinda Gabbe

**Affiliations:** a School of Public Health and Preventive Medicine, Monash University, Melbourne, Victoria, Australia; b Monash Department of Surgery, The Alfred, Melbourne, Victoria, Australia; c Department of Orthopaedic Surgery, The Alfred, Melbourne, Victoria, Australia; d Monash Centre for Health Research and Implementation, Monash University, Melbourne, Victoria, Australia; e Department of Orthopaedics, Royal Melbourne Hospital, Parkville, Victoria, Australia; f Population Data Science, Swansea University Medical School, Swansea University, Swansea, Wales, United Kingdom

**Keywords:** Cauda equina syndrome, Injury, Trauma, Spine, Fracture, Outcomes, Incidence, Bowel, Bladder

## Abstract

**Background:**

Little is known about the incidence, management or long-term outcomes of traumatic cauda equina syndrome (CES), with few cohort studies. The purpose of this study is to establish the incidence and long-term outcomes of traumatic CES in Victoria, Australia. This study is a registry-based cohort study, and people with a diagnosis of traumatic CES from 2010 to 2022 were recruited from the Victorian State Trauma Registry.

**Methods:**

An incidence rate was calculated by dividing the amount of new cases each year by the estimated population in Victoria. Demographic, injury and hospital admission details were extracted from the Victorian State Trauma Registry. Routine follow-up occurred at 6, 12- and 24- months postinjury, with a focus on health-related quality of life outcomes using the EuroQol EQ-5D scale, level of disability using the World Health Organization Disability Assessment Schedule (WHODAS) score and return to work outcomes. An additional telephone interview undertaken at a median 6.8 years postinjury collected the EQ-5D, bowel and bladder outcomes. Descriptive statistics were used to analyse data. Mixed effects regression modelling was used to model change in EQ-5D outcomes over time.

**Results:**

Of the 94 participants, most were men (67%), the median age at injury was 41 years, and the most common cause was road trauma (35%). The incidence rate ranged from 0.56 to 2.51 per million per year. Most people reported problems on the EQ-5D at all 4 follow-up time points, with no clear improvement over time. 47% of people had not returned to work 24 months after injury. Of the survivors who completed the additional follow-up, 41% of people experienced constipation and 51% reported almost losing bladder continence at least once per week.

**Conclusions:**

While the incidence rate of traumatic CES was low, most people experienced long-term sequelae, highlighting the impact of this injury on peoples’ lives. Multijurisdictional studies may be needed to comprehensively measure the impacts of this injury.

## Introduction

The cauda equina is the continuation of the spinal nerve roots after the spinal cord terminates at the L1-2 vertebral level, innervating the pelvis, perineum, lower limbs, bowel, bladder and reproductive organs.^1^^,^^2^ Cauda Equina Syndrome (CES) occurs when the cauda equina spinal nerve roots are compressed or disrupted, causing a constellation of clinical features, such as bowel, bladder and sexual dysfunction, as well as sensory and motor changes. Whilst CES can have a variety of aetiologies, traumatic CES occurs when this syndrome arises after a traumatic injury.^3^ While the incidence rate of traumatic spinal cord injury in Australia is reported as between 21 and 32 per million per year, the incidence rate of traumatic CES is unknown.^4^ Traumatic CES has been described in case reports and case series as causing long term neurological sequelae.^5^^,^^6^ A few cohort studies exist, with a primary focus on ambulation outcomes following injury to the cauda equina. A retrospective study by Brouwers et al.^7^ comparing outcomes at varying levels of spinal cord injury had a subgroup of 306 patients with traumatic CES, and described improvement in ambulatory, bowel and bladder outcomes between discharge and at 1 year follow up. Similar improvement in ambulation, bowel and bladder outcomes were demonstrated between admission and hospital discharge by a prospective cohort study by Attabib et al.,^8^ consisting of 214 patients with traumatic CES. The bowel and bladder outcomes in both papers were reported using the Spinal Cord Independence Measure Score (SCIMS), with Attabib et al. also using the Functional Independence Measure (FIM) Score, which are tools that measure a person's ability to independently manage their bowel or bladder. Neither the FIM score or the SCIMS score directly measure the level of neurological dysfunction, rather reflecting a person's ability to independently manage their symptoms. Furthermore, the SCIMS sphincter subscore reports bladder, bowel and respiratory outcomes as a single outcome, obscuring the nature of recovery of each organ system independently. Overall, the prognosis of bowel and bladder outcomes remain unclear beyond 1 year after injury, with no truly appropriate scoring system being used to capture neurological recovery in this patient population. Despite being a life-long injury, little is known about recovery in the long-term. The aims of this study were to establish the incidence rate of traumatic CES in Victoria, Australia, and to describe the long-term outcomes of this injury.

## Methods

### Setting

Victoria is the second most populous state in Australia, with a population of 6.8 million.^9^ The Victorian State Trauma System was established in the year 2000, and includes 138 trauma receiving hospitals, with 3 hospitals considered as major trauma services.^10^ Ambulance transport and retrieval is provided by Ambulance Victoria, with patients triaged to trauma hospitals according to established guidelines.^11^ The Victorian State Trauma Registry, established in 2001, is a population-based trauma registry which routinely collects data about all major trauma patients managed in the trauma system.^12^

### Participants and procedures

The study population included people aged 16 years and over with an Abbreviated Injury Scale (AIS), or the International Statistical Classification of Diseases and Related Health Problems, Tenth Revision, Australian Modification (ICD-10-AM), code for cauda equina injury, and a date of injury from January 2010 to December 2021 (inclusive) (Table 1). To assign an AIS code for CES, the treating team must diagnose the patient with CES, alongside surgical, radiographic or autopsy evidence.^13^ The ICD-10-AM code is assigned by health coders, based on the diagnosis established by the treating team from the patient clinical health records.^14^ Whilst no set criteria is used in the Victorian setting to diagnose CES, it is often diagnosed based on a constellation of clinical features including bowel, bladder, sexual, sensory and motor changes presenting in the setting of possible spinal injury and supporting radiographic evidence.^3^ Ethics approval for this project was granted both by the Alfred Hospital Human Research Ethics Committee (project number 165/23) and Monash University Human Research Ethics Committee (project number 37956).

**Table 1:** AIS and ICD-10-AM injury codes.

AIS Cauda equina injury codes	
630600.3	Cauda equina contusion, NFS.
630602.3	Cauda equina contusion with transient neurological signs but NFS as to fracture/dislocation.
630604.3	Cauda equina contusion with transient neurological signs with no fracture/dislocation.
630606.3	Cauda equina contusion with transient neurological signs with fracture.
630608.3	Cauda equina contusion with transient neurological signs with dislocation.
630610.3	Cauda equina contusion with transient neurological signs with fracture and dislocation.
631620.3	Incomplete cauda equina syndrome but NFS as to fracture/dislocation.
630622.3	Incomplete cauda equina syndrome with no fracture or dislocation.
630624.3	Incomplete cauda equina syndrome with fracture.
630626.3	Incomplete cauda equina syndrome with dislocation.
630628.3	Incomplete cauda equina syndrome with fracture and dislocation.
630630.4	Complete cauda equina syndrome but NFS as to fracture/dislocation.
630632.4	Complete cauda equina syndrome with no fracture/dislocation.
630634.4	Complete cauda equina syndrome with fracture.
630636.4	Complete cauda equina syndrome with dislocation.
630638.4	Complete cauda equina syndrome with fracture and dislocation.

ICD-10-AM Cauda equina injury codes	

S34.3	Injury of cauda equina.

AIS, Abbreviated Injury Score; ICD-10-AM, International Statistical Classification of Diseases and Related Health Problems, Tenth Revision, Australian Modification; NFS, not further specified.

Demographic characteristics, injury event details, hospital stay variables and patient-reported outcomes were extracted from the registry. Participants’ residential address postcodes were classified as either metropolitan or regional, as determined by the Accessibility/Remoteness Index of Australia (ARIA), and assigned an Index of Relative Socio-Economic Advantage and Disadvantage (IRSAD), with one reflecting the most disadvantaged areas, and 5 representing the most advantaged areas.^15^^,^^16^ The Charlson Comorbidity Index (CCI) assigns a numerical value to a patient's mortality risk, based on whether they have any existing comorbid diseases, with a score of zero indicating no comorbidities, and higher scores equating to a higher risk of dying from their respective comorbidities.^17^ ICD-10-AM diagnosis codes were mapped to CCI conditions to calculate the CCI. The Injury Severity Score (ISS), ranging from 1 to 75 reflects the severity of a patient's injuries, with a score over 12 considered major trauma.^18^

Patient reported outcomes are routinely collected via telephone interview by registry staff at 6-, 12- and 24-months post injury. Several attempts are made before participants are considered lost to follow-up. Proxies are used in cases where participants are unable to complete the interview or where it is the participant's preference. Routine linkage with the Victorian Registry of Deaths, Births and Marriages is undertaken to identify postdischarge deaths. At the routine 6-, 12- and 24-months follow-up, the EuroQol 5-level 5-dimension questionnaire (EQ-5D-5L), the World Health Organization Disability Assessment Schedule 2.0 (WHODAS) and return to work outcomes are collected.

The EuroQol EQ-5D-5L is a widely used quality of life measure, which examines the domains of mobility, self-care, usual activities, pain and/or discomfort, and anxiety and/or depression.^19^ Participants respond as experiencing no, slight, moderate, severe or extreme problems to each domain. The registry used the 3-level version (EQ-5D-3L) prior to July 2018. Therefore, for this study, the 5L was mapped to the 3L version. Responses to each dimension are used to create unique health states. Each unique health state is given a utility score, based on societal values as to which health states are considered worse than others, with Australian specific tariffs applied.^20^ A negative utility score represents a state worse than death, a score of zero represents a health state equivalent to death and one represents perfect health.^21^ Return to work or study outcomes are also routinely collected by the registry for people who were previously working or studying prior to their injury. These include whether the person has returned to work/study (yes/no), whether they have returned to the same organizations or workplace (yes/no) and whether they are working in the same role at that organization (yes/no).

The WHODAS is an outcome measure examining 6 disability dimensions, including: cognition, mobility, self-care, social interaction, general life activities and social participation. People are asked to score 12 questions that pertain to the last 30 days. Item scores are summed to provide an overall score between zero and 48. The total WHODAS score was categorised as “<10”, and “10+”, with the latter category representing more severe disability.^22^

In addition to the existing outcomes data captured by the registry, an additional telephone interview was carried out over May and June 2023 to collect the EQ-5D-5L, and bladder and bowel outcomes. Bladder and bowel outcomes are not routinely collected by the registry at the 6-, 12- and 24-months routine follow-up. People previously lost to follow-up at the 3 previous time points were not contacted. Six contact attempts were allowed before a person was considered lost to follow-up.

An existing Multiple Sclerosis bowel and bladder outcomes tool, developed by the National Multiple Sclerosis Society Consortium of Multiple Sclerosis Centers, was adapted in the absence of a bowel and bladder outcome tool specifically designed for CES.^23^ People were asked how many times a week they were fecal/urinary incontinent, how many times they almost lost control of bowel or bladder, the number of times they had to alter activities due to bowel or bladder problems, and the number of times they were constipated. People were also asked to rate from a scale of 0 (no restructuring) to 10 (most severe restructuring), how much they had restructured their life due to bowel and bladder symptoms. People were also asked to rate on a scale of 0 (no change) to 10 (all issues resolved) how much their bowel and bladder control has improved since injury.

A form created in REDCap was used to collect the additional telephone interview questionnaire and data by registry staff.^24^^,^^25^ Stata Version 17 (StataCorp, College Station, TX) was used for statistical analysis. All analysis was performed in the Monash Secure eResearch Platform (SeRP).

### Data analysis

The data were summarized using descriptive statistics. The incidence rate was calculated by dividing the number of new traumatic CES cases each year by the number of people aged 16 years and above who lived in Victoria at the 30th of June for that year, sourcing Victorian population figures from the estimated resident population as calculated by the Australian Bureau of Statistics.^26^ For each incidence rate, 95% confidence intervals were calculated.

For categorical variables, frequencies and percentages were used. Continuous variables were summarized using the mean and standard deviation (SD) if normally distributed, or median and interquartile range (IQR) otherwise. Mixed effects regression modelling was also used to model EQ- 5D outcomes over time to account for the correlation between multiple responses from the same individual (repeated measures). Predicted proportions and 95% confidence intervals at each time point postinjury were calculated. Due to the relatively small study cohort, multivariable modelling was not done.

## Results

### Cohort overview

**Fig. 1:**
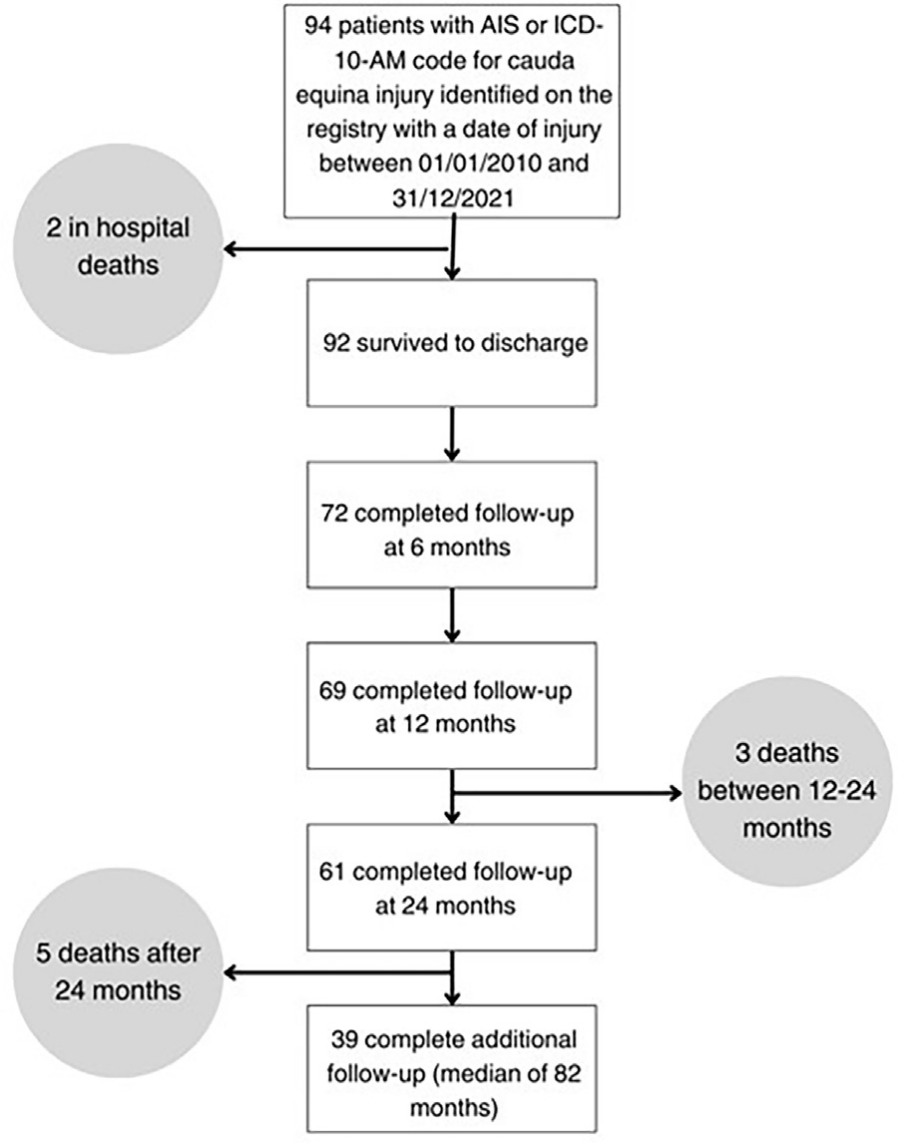
Flowchart of patient follow-up.

**Fig. 2:**
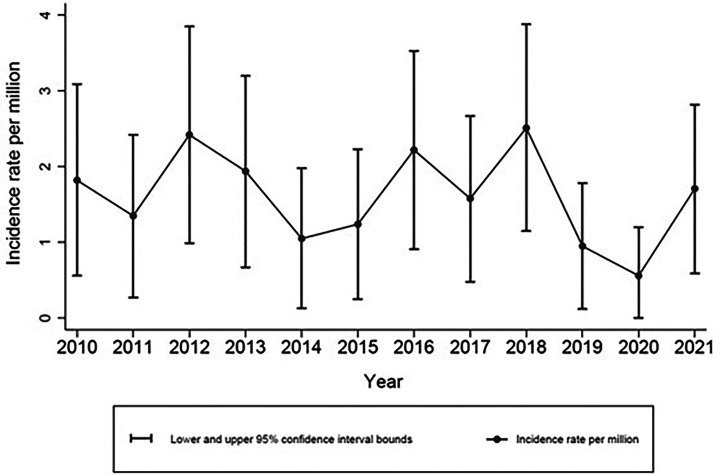
The incidence rate of traumatic CES in Victoria 2010–2021.

Ninety-four people with traumatic CES were recorded on the Victorian State Trauma Registry in the timeframe; 92 survived to hospital discharge (Fig. 1). Of the 94 people, 67% were male, and the median age was 41 years. The most common causes were road traffic crashes and high falls (>1 m) (Table 2). Concomitant lumbar fracture was more prevalent than sacral fracture, and 61% of patients received spinal decompression surgery during their initial hospital stay. The incidence ranged from 0.56 per million per year to 2.51 per million per year between the years of 2010 and 2021, with the rate of injury staying similar over time (Fig. 2).

**Table 2:** Demographics table of N=94 patients.

Variable		N (%)
Age category	17-39 y	46 (49%)
	40-59 y	25 (27%)
	60+ y	23 (24%)
Sex	Male	63 (67%)
	Female	31 (33%)
Level of education attained*	Did not complete high school	25 (36%)
	Advanced diploma	18 (26%)
	Completed high school	11 (16%)
	University	16 (23%)
Metropolitan or regional area of residence†	Metropolitan area	63 (70%)
	Regional area	27 (30%)
Socioeconomic Status (IRSAD)‡	1 (most disadvantaged)	10 (11%)
	2	17 (19%)
	3	17 (19%)
	4	24 (27%)
	5 (least disadvantaged)	22 (24%)
Charlson Comorbidity Index weight (CCI)	0	62 (66%)
	1	21 (22%)
	>1	11 (12%)
Self-reported disability prior to injury§	No	62 (81%)
	Yes	15 (19%)
Cause of injury	Road trauma	33 (35%)
	High falls	29 (31%)
	Low falls	21 (22%)
	Other	11 (12%)
Injury Severity Score > 12	No	26 (28%)
	Yes	68 (72%)
Concomitant sacral fracture	No	71 (76%)
	Yes	23 (24%)
Concomitant lumbar fracture	No	16 (17%)
	Yes	78 (83%)
Any spinal decompression surgery during hospital stay║	No	32 (34%)
	Yes	57 (61%)
Length of hospital stay	<7 days	25 (27%)
	7-13 days	28 (30%)
	14-20 days	15 (16%)
	21-27 days	12 (13%)
	>27 days	14 (15%)
Intensive care unit stay	No ICU stay	74 (79%)
	ICU stay	20 (21%)
Discharge destination	Rehabilitation	55 (59%)
	Home	27 (29%)
	Hospital for Convalescence	6 (6%)
	In hospital death	2 (2%)
	Other	4 (4%)

CCI, Charlson Comorbidity Index; ICU, intensive care unit; IRSAD, Index of Relative Socio-economic Advantage and Disadvantage.

⁎ n=24 missing.

† n=4 missing.

‡ n=4 missing.

§ n=17 missing.

║ n=5 missing.

### Health status and return to work

Of the survivors, 78% completed follow-up at 6 months, 75% at 12 months, 69% at 24 months and 46% at the additional follow-up (Fig. 1). The additional follow-up took place at a median time of 6.8 years (82 months), with an IQR of 4.8 to 11.1 years.

**Fig. 3:**
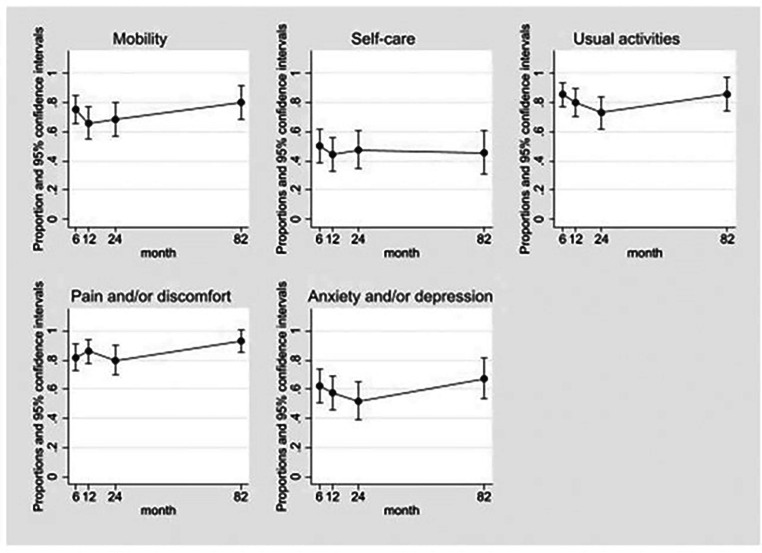
EQ-5D dimensions adjusted predictions mixed effect regression modelling graphs.

Within the EQ-5D dimensions, problems with pain and/or discomfort were more prevalent across the time points. Whilst a higher percentage of participants experienced problems at the additional follow-up compared to at 24 months in most dimensions (Table 3), there was no clear evidence of change on the EQ-5D items over time (Fig. 3).

**Table 3:** EQ-5D and return to work outcomes at follow-up.

Follow-up time point Total respondents (N)	6 mo N=72	12 mo N=69	24 mo N=58	Median of 6.8 y N=39
EQ-5D dimensions				
Mobility				
No problems	17 (24%)	25 (36%)	20 (34%)	8 (21%)
Some degree of problems	55 (76%)	44 (64%)	38 (66%)	31 (79%)
Self-care				
No problems	35 (49%)	39 (57%)	31 (53%)	22 (56%)
Some degree of problems	37 (51%)	30 (43%)	27 (47%)	17 (44%)
Usual activities				
No problems	10 (14%)	14 (20%)	15 (26%)	5 (13%)
Some degree of problems	62 (86%)	55 (80%)	43 (74%)	34 (87%)
Pain and/or discomfort				
No problems	13 (18%)	10 (14%)	12 (21%)	3 (8%)
Some degree of problems	59 (82%)	59 (86%)	46 (79%)	36 (92%)
Anxiety and/or depression*				
No problems	27 (38%)	30 (43%)	29 (51%)	16 (41%)
Some degree of problems	44 (62%)	39 (57%)	28 (49%)	23 (59%)
EQ-5D mean utility score				
Utility score mean	0.44	0.50	0.52	
(95% Confidence Interval)	(0.35, 0.53)	(0.42, 0.59)	(0.43, 0.62)	
Return to work outcomes †				
Returned to work	17 (38%)	18 (43%)	19 (53%)	
Did not return to work	28 (62%)	24 (57%)	17 (47%)	

Missing values:

⁎ N=1 did not answer the anxiety and/or depression section at 6 months and at 24 months.

† In the return to work outcomes, N=27 did not respond at 6 months, N=27 did not respond at 12 months and N=25 did not respond at 24 months.

The EQ-5D utility score improved over time, suggesting overall health perception improved between 6 and 24 months (Table 3). The percentage of people scoring more than 10 on the WHODAS increased between 6 and 24 months, with 65% at 6 months, 59% at 12 months and 68% at 24 months. Forty-seven people were working for income or studying prior to injury, with return to work rates increasing over time; 47% had not returned to work 24 months postinjury (Table 3).

### Bowel and bladder outcomes

At the additional follow-up, patients reported severe bladder continence issues, with 6 people reporting daily loss of bladder control, 9 reporting almost losing control of bladder daily, and 6 reporting having to alter their activities on a daily basis due to bladder problems. Overall, less people experienced residual bowel sphincter problems than with bladder sphincter problems, with 8 patients experiencing persistent bowel incontinence compared to 14 patients experiencing persistent bladder incontinence (Table 4).

**Table 4:** Frequency of bladder and bowel problems experience by patients during the week at a median of 6.8 years postinjury.

	Not at all	At least once per wk
Bladder outcomes		
No. of times lost control of bladder or had an accident N (%)	25 (64%)	14 (36%)
No. of times almost lost control of bladder or had an accident N (%)	19 (49%)	20 (51%)
No. of times altered activities because of bladder problems N (%)	32 (82%)	7 (18%)
Bowel outcomes		
No. of times they experienced constipation N (%)	23 (59%)	16 (41%)
No. of times lost control of bowels or had an accident N (%)	31 (79%)	8 (21%)
No. of times almost lost control of bowels or had an accident N (%)	27 (69%)	12 (31%)
No. of times altered activities because of bowel problems N (%)	32 (82%)	7 (18%)

**Fig. 4:**
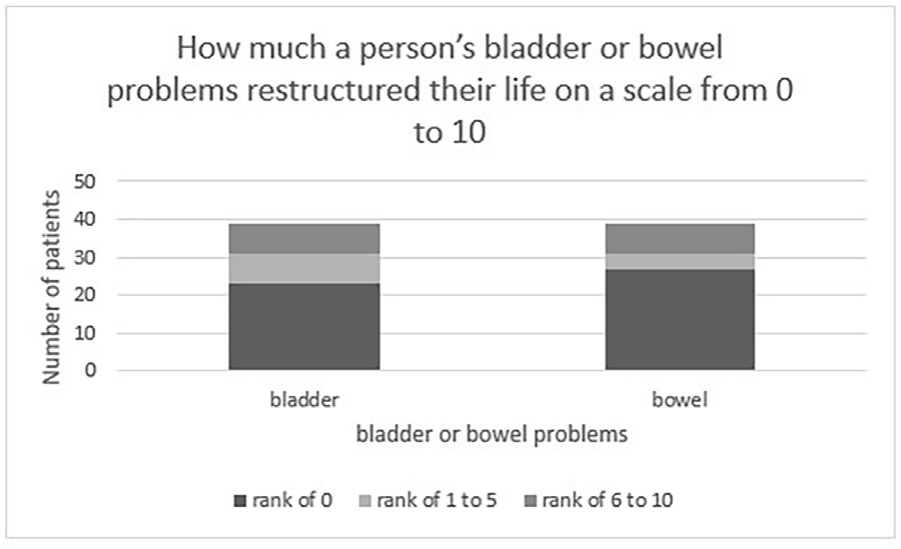
Bowel and bladder impact on a person's life graph.

**Fig. 5:**
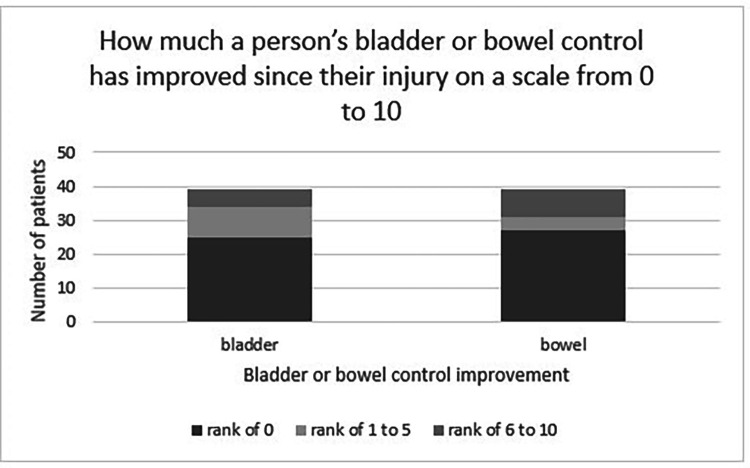
Bowel and bladder symptom improvement since injury graph.

On a scale from zero to 10, 69% of patients reported their bowel symptoms as impacting their life as zero compared to 59% who reported their bladder symptoms as impacting their life as zero (Fig. 4). However, in the group that did report restructuring of their life, there was a higher degree of severity, as 6 people reported 10 out of 10 severity in either bowel or bladder impacts. Most respondents scored their improvement on bladder symptoms (64%) and bowel symptoms (69%) over time as zero (no improvement), whereas 3% reported full resolution of bladder symptoms, and 10% of bowel symptoms (Fig. 5).

## Discussion

The study in this article was the first study to examine quality of life, return to work, bowel, and bladder outcomes of traumatic CES over the long-term, and enable the calculation of its incidence. A low incidence rate confirms CES as a rare injury. The epidemiological profile of the people was consistent with major trauma in Victoria and the literature.[5], [6], [7]^,^[27], [28], [29], [30], [31], [32], [33], [34], [35], [36], [37], [38], [39], [40], [41], [42], [43], [44], [45], [46], [47], [48], [49], [50], [51], [52], [53], [54] Despite low energy mechanisms not being extensively reflected in the literature, the study in this article revealed that 22% of people recorded a low fall as the cause of injury.[55], [56], [57] Another key finding was that concomitant lumbar fracture was more prevalent than sacral fracture.

Problems within each of the EQ-5D domains remained prevalent at a median of 6.8 years postinjury. Whilst we observed little evidence of improvement over time within the EQ-5D dimension of self-care, a retrospective study by Brouwers et al.,^7^ featuring a cohort of traumatic CES patients, described a significant improvement in self-care 1-year post injury using the SCIMS. In our study, 79% reported difficulty with mobility at the additional follow up with no clear improvement over time, which contrasts to retrospective cohort studies by Brouwers et al. and Attabib et al., demonstrating motor function improvement over time between baseline and at 12-months postinjury and hospital discharge respectively.^7^^,^^8^

The low number of participants precluded adjustment for confounders and subgroup comparison. It is possible that participants at the additional time point had more severe problems, prompting their participation in additional follow-up. Improvement in utility scores over a 24-month period may indicate people learning to live with their injury despite ongoing problems. While the proportion of patients who had returned to work or study increased at 24 months postinjury, 47% had not returned to work. The return to work outcomes is similar to the prevalence described in a meta-analysis of return to work outcomes following spinal cord injury published in 2022, with a reported prevalence of 45.8%.^58^

It is clear a certain proportion of people still experienced issues with bowel and bladder at a median follow-up of almost 7 years. Whilst most participants in our study report no improvement in bowel (69%) or bladder (64%) function over time, this contrasts to findings by other studies showing bowel and bladder improvement over time.^7^^,^^8^ Direct comparison to these studies is challenging, as the tools used to measure bladder and bowel outcomes focus on management, rather than quantifying sphincter dysfunction. A traumatic CES subgroup from a cohort study by Brouwers et al. used the Spinal Cord Independence Measure Score, and described an improvement in the sphincter and respiratory management subscore at 12-months compared to baseline, which reports bowel, bladder and respiratory management together as a single outcome measure.^7^ Another traumatic CES cohort study comparing outcomes between admission and hospital discharge by Attabib et al.^8^ used the Functional Independence Measure score, and showed improvement at discharge in a person's ability to manage their bladder and bowel.

The study in this article is a unique contribution to the literature, in that it is one of very few cohort studies to investigate traumatic cauda equina syndrome as a stand-alone injury. The use of multiple follow-up time points enabled a more comprehensive account of trends in outcomes over a substantial length of time, and a median additional follow-up time close to 7 years is a substantially longer period of follow-up than previous traumatic CES studies to our knowledge. The use of a detailed and specific bowel and bladder questionnaire, whilst not specific to traumatic CES and was adapted from a Multiple Sclerosis tool, enabled an accurate reflection of sphincter challenges experienced by people, which may be understated using other tools in previously published studies. This study is also the first of this size to include overall quality of life measurements and return to work outcomes for this cohort of patients. To our knowledge, this is the first population incidence rate reported of this syndrome due to traumatic cause.

Despite being a small cohort, this study encapsulates the entire adult population with traumatic CES over the years of 2010-2021 in Victoria, Australia. However, the incidence rate may be an underestimate, with potentially missed cases due to the absence of a universal diagnostic criteria for CES where it is at the discretion of the treating team to make a diagnosis, upon which the AIS and ICD-10-AM codes are based.^3^^,^^59^ Furthermore, late or missed diagnoses of traumatic CES, of which there a several case reports in the literature, may also not be included in the registry.^43^^,^^44^^,^^57^^,^^60^ The use of the EQ-5D scale and subsequent ability to generate utility scores provides a standardized method to measure global health outcomes. In regards to data reliability, the registry data has a high level of completeness with an opt-out rate of less than 0.4 percent, with strict validation rules, trained coders and reference manuals. Whilst the bowel and bladder questionnaire were based on a validated tool used to quantify outcomes in patients with Multiple Sclerosis, reliability and validity checks were not done on this tool for this study. A limitation of the study described in this article was the notable loss to follow-up, raising the potential for responder bias in the final interview. Another limitation of this study was that certain outcomes were not explored, such as sexual dysfunction and sensory changes, with these outcomes being reported in the literature.^44^^,^^48^^,^^53^^,^^61^^,^^62^

## Conclusion

This cohort study showed that the incidence of traumatic CES in Victoria was low, and closely mirrors the broader trauma cohort in Victoria. It was revealed that most people experienced long-term sequelae following their injury. Further multijurisdictional cohort studies and qualitative research may be done to further investigate this injury.

## Funding

The Victorian State Trauma Registry (VSTR) is funded by the Department of Health, State Government of Victoria and the Transport Accident Commission. Belinda Gabbe was supported by a 10.13039/501100000925National Health and Medical Research Council of Australia Investigator Grant (ID 2009998).

## Declaration of Competing Interests

One or more of the authors declare financial or professional relationships on ICMJE-NASSJ disclosure forms.
